# A case for increasing taxes on cigarettes, vapes and oral nicotine pouches, Kenya

**DOI:** 10.2471/BLT.23.290985

**Published:** 2024-07-04

**Authors:** Cyprian M Mostert, Olalekan A Ayo-Yusuf, Manasi Kumar, Andrew Aballa, Willie Njoroge, Edna Bosire, Linda Khakali, John Thomi, Karambu Muthaura, Lukoye Atwoli, Zul Merali

**Affiliations:** a Department of Population Health, Aga Khan University, 3rd Parklands Avenue off Limuru Road, P.O. Box 30270, Nairobi 00100, Kenya.; b Africa Centre for Tobacco Industry Monitoring and Policy Research, University of Pretoria, School of Health Systems and Public Health, Pretoria, South Africa.; c Institute for Excellence in Health Equity, New York University School of Medicine, New York, United States of America.; d Brain and Mind Institute, Aga Khan University, Nairobi, Kenya.; e National Taxpayers Association, Nairobi, Kenya.; f Policy and Tax Advisory Division, Kenya Revenue Authority, Nairobi, Kenya.

Kenya became a party to the World Health Organization (WHO) Framework Convention on Tobacco Control (FCTC) in 2004; three years later, the country enacted the Tobacco Control Act. The FCTC and this national policy advocate for progressive cigarette taxation to reduce cigarette consumption. However, the current Kenyan cigarette tax regime fails to control cigarette consumption efficiently, especially among young people. For example, the 2007 Global Youth Tobacco Survey (13–15 years) revealed that 1 out of 10 students aged 13 to 15 years were current smokers, and boys were twice as likely to be using tobacco than girls.^1^^,^^2^ In 2013, WHO reported that this prevalence estimate remained relatively unchanged despite the adoption of the Tobacco Control Act. To date, no comparable survey has been published in Kenya, but in 2022, preliminary findings of a study conducted by the Kenya Tobacco Board on the use of tobacco and its products in four counties showed that consumption of e-cigarette and nicotine pouches was increasing among young people in Kenya.^3^ These developments underscore the need for reforming tax policies to protect young Kenyans from nicotine- and tobacco-related harms.

## Implementation complexity 

Kenya decentralized the implementation of the Tobacco Control Act to 47 county governments in 2010. However, this decentralization has created a need for more institutional coherence in implementing the act. The tobacco industry exploits these fragmented counties by targeting the young people with tobacco products, particularly in the eastern, central and coast counties,^4^ where less resources are available for governance and control of substance use. In this context, the health ministry, with the support of WHO and academic institutions, should collect data and analyse the implementation of excise duties and other reforms in different jurisdictions to capture insights and ways to address the gaps of the existing fragmented administrative system. The Tobacco Control Act is being implemented differently in the 47 counties, and it is the collective responsibility of all stakeholders to ensure institutional coherence in the implementation of the act. The central government needs to enforce measures that ensure homogeneous implementation of tobacco control in all counties.

## Tobacco tax policy 

In 2023, the average retail price for 20-pack cigarettes in Kenya was 340 Kenyan shillings (KES) (approximately 2.59 United States dollars, US$). Cigarette taxes constitute about one third of the retail price,^5^ which is substantially lower than WHO’s recommendation that taxes should account for 70% to 75% of the retail price to reduce cigarette consumption. Because smoking in Kenya is linked to economic status, undertaxed cigarettes are more affordable to a higher proportion of the Kenyan population,^2^ aggressive taxation on cigarettes is needed.

In 2021, Kenya scored 0.88 out of 5.00 on the economics of tobacco (also called tobacconomics) tax scorecard.^4^ A score of 5.00 represents the best tobacco tax regime that reduces tobacco consumption. Kenya’s score is lower than the average score for middle-income countries (1.78), and lower than the average score for lower-middle-income countries (1.42) and that of the African continent (1.36).^4^

The lobbying power of the tobacco industry blunts the effectiveness of the cigarette excise tax policy. In 2017, the industry lobbied the Kenyan government to adopt a two-tier tax system,^4^ which caused differential taxes between cheap and premium cigarettes. Such a tax system induces substitution effects, incentivizing smokers who cannot afford premium cigarettes to downgrade to cheaper cigarettes instead of quitting. The same two-tier tax system makes it more affordable for young people to initiate smoking using cheaper cigarettes.

Despite the failures of Tobacco Control Act in reducing the prevalence of tobacco use among young people in Kenya,^1^ no active steps have been taken to reform the cigarette excise tax policy. If business continues as usual, Kenya will incur significant – yet preventable – social and economic costs from tobacco-related illnesses. The country has about 9000 tobacco-related deaths annually, and loses approximately KES 40 billion (US$ 307 million) yearly^6^ due to tobacco-related conditions – resources that could be used to advance human development.

## Oral nicotine taxation 

As any other industry, the tobacco industry wants to increase its customer base. This intention is evidenced by the recent launch of nicotine pouches in the Kenyan tobacco market, and all indications are that the use of nicotine pouches will increase over time.

The tobacco industry consistently claims that modern oral nicotine pouches are safer than combustible cigarettes^7^ and support smoking cessation. However, no independent study has confirmed these claims. On the contrary, some scholars have suggested that nicotine pouches are a gateway to increased cigarette smoking via nicotine habituation,^8^ and European studies have rejected the view that nicotine pouches are effective instruments for smoking cessation.^9^ In its strategy for global nicotine reduction, WHO highlighted that novel nicotine pouches could double the risk for non-smokers to start smoking cigarettes. Therefore, policy-makers in Kenya must not buy into industry claims without subjecting them to a proper independent investigation into the impact of oral nicotine pouch use on smoking initiation.

In Kenya, the tobacco industry is using the claim of safety to demand favourable treatment from the tax authorities, arguing that modern oral nicotine pouches should attract a lower excise tax rate than the lowest tax tier for cigarettes.^7^ So far, the current trading price of a nicotine pouch (10 mg) in Kenya is KES 350 (US$ 2.61), of which less than KES 0.02 are the tax on this product.

Such negligible taxation is unacceptable, considering international studies show that modern oral nicotine pouches have alarmingly high nicotine levels^9^ and come in flavours that are attractive to young people. Children from primary school (younger than 13 years) are being introduced to tobacco products, with recent statistics showing that about 3% of primary school pupils in Kenya are currently using tobacco.^10^

Unfortunately, the current Kenyan tax does not help reduce nicotine pouch consumption and fails to prevent young people from starting. Therefore, a case for reforming the excise tax policy on cigarettes and nicotine products exists; many countries are exploring this option, and WHO is needed to guide these processes.

## Taxation of vapes 

The National Treasury of Kenya set the tax on e-cigarette liquids (e-liquids) at KES 70/mg (US$ 0.5/mg) in 2022. Therefore, the tax per 50 mg should be KES 3500 (US$ 26). So far, there are no 50 mg e-cigarette liquids in the Kenyan online market that are trading above KES 3000 (US$ 22). Such low prices reflect inadequate taxation oversight, as e-liquids are being sold below the tax value. 

The government needs to coordinate robust and comprehensive operations to regulate the entire supply chain of the e-liquid market. These measures should be combined with targeted cessation support and awareness campaigns focusing on vulnerable young people.

## Lessons from Uganda

Kenya can do better by replicating Uganda’s approach, which taxes tobacco progressively. The tax scores for Uganda increased between 2014 and 2020 from 0.63 to 1.25, and the country currently has a tax share of the retail price of 41%.^11^ In contrast, tax scores for Kenya dropped from 1.13 in 2014 to 0.88 in 2020 and the country’s current tax share of the retail price is 39%. Furthermore, the age-standardized tobacco use prevalence decreased in Uganda by 10.4 percentage points between 2005 and 2020 (from 18.8% to 8.4%). This decrease was greater than in Kenya, which only saw a 5.7 percentage points decrease over the same period (from 16.8% to 11.1%).^12^ Indeed, there is space for higher taxation in Kenya to reduce tobacco consumption.

## Conclusion

For the Kenyan government to succeed in reducing smoking and nicotine addictions in young people, it needs to take four steps in tax reform. First, implement aggressive tobacco taxation in line with the WHO FCTC. Second, eliminate the two-tier tax system. Third, introduce high taxation of nicotine pouches or ban these products to reduce consumption. Fourth, regulate e-liquids. 

The current lack of up-to-date data on the key characteristics of users, particularly disaggregated by wealth quintiles, age, gender and urban versus rural population, is an important gap that needs to be filled. Consistent data collection to monitor cigarette, vape and oral nicotine pouches use in the youth population is essential, especially at county level. Such data will provide a solid evidence base and play a pivotal role in informing targeted, strategic policies and programmes. We strongly recommend generating these data to improve targeted policy interventions aimed at preventing tobacco and nicotine product consumption.

Finally, a social justice tax framework is needed to hold the industry accountable for these public health consequences. Working with civil society and governments, WHO through its normative standard for tobacco control may need to develop such a tax framework, and countries should present punitive taxation measures for industry interference. All countries stand to benefit from such a framework.
